# Upper border of anal transitional zone: a new landmark for endoscopic internal hemorrhoid therapy

**DOI:** 10.3389/fmed.2026.1903391

**Published:** 2026-08-27

**Authors:** Jingyao Li, Huiyan Li, Hao Xu, Jing Dong, Tiantian Guo, Hongjiao Yao, Qi Yang

**Affiliations:** Department of Gastroenterology, The Affiliated Hospital of Northwest University (Xi’an No.3 Hospital), Xi’an, China

**Keywords:** anal transitional zone, endoscopy, free nerve endings, internal hemorrhoids, rubber band ligation, sclerotherapy

## Abstract

**Background and aim:**

Endoscopic therapies are important approaches for managing internal hemorrhoids. This study assessed the feasibility of endoscopic examination of the upper border of anal transitional zone (ATZ) and its clinical utility and underlying anatomical basis as a landmark for internal hemorrhoid.

**Methods:**

Mucosal demarcation line (MDL) and the dentate line were identified and measured in 207 adults using white-light and narrow band imaging (NBI) endoscopy, with three biopsies for histologic confirmation. Five anorectal specimens were examined employing hematoxylin and eosin staining and immunohistochemistry for S100 and PGP9.5 to evaluate hemorrhoidal cushion position and nerve distribution. Ninety-three patients undergoing sclerotherapy or rubber band ligation (RBL) with MDL as reference were followed using symptom questionnaires and complication assessments.

**Results:**

(1) A distinct MDL was identified above the dentate line in 69.08% of participants under white light imaging, increasing to 96.14% under NBI (*p* < 0.001), located 8.18 ± 2.96 mm above the dentate line. (2) Histologically, MDL represented the upper border of ATZ based on epithelial transition; hemorrhoidal cushions extended 6.81 ± 4.21 mm above MDL. (3) PGP9.5-positive intraepithelial free nerve endings were present within ATZ at a lower area ratio than the non-keratinized (*p* = 0.038) and keratinized mucosa below the dentate line (*p* < 0.001) and were absent in the rectal mucosa. (4) Using MDL (the upper border of ATZ) as reference, six-month effective rates were 91.80% for sclerotherapy and 93.75% for RBL, with significant improvement in anal discomforts and defecatory disorders. Postoperative discomfort occurred in 6.56 and 18.75% of patients, respectively, without major complications.

**Conclusion:**

The upper border of ATZ can serve as a reliable endoscopic landmark for safe and effective treatment of internal hemorrhoid therapy, likely due to its anatomical relationship with the hemorrhoidal cushions and preservation of the ATZ.

## Introduction

Internal hemorrhoids are among the most common anorectal disorders (1) and are characterized by hematochezia, mucosal prolapse, perianal moisture, pruritus, sensation of anal fullness, and defecatory disturbances (2, 3). The pathophysiology of symptomatic internal hemorrhoids involves abnormal displacement of the anal cushions due to connective tissue degradation, accompanied by vascular dilation and blood stasis within the hemorrhoidal plexus. Current clinical guidelines from multiple countries recommend endoscopic therapies, particularly sclerotherapy and rubber band ligation (RBL), as important nonoperative treatments for symptomatic internal hemorrhoids (4–6).

Accurate identification of operative landmarks is critical for the safe and effective endoscopic management of internal hemorrhoids. Guidelines from the United States and China recommend avoiding the dentate line and the distal anal canal during procedures to prevent complications (4, 7). Inadvertent sclerotherapy or ligation below the dentate line can result in severe postoperative perianal pain. However, there is no consensus regarding the optimal treatment site above the dentate line, which often depends on operator’s experience. Even when the dentate line is used as a reference, postoperative discomfort including pain, fullness, and defecatory or urinary difficulty remains common, particularly after RBL (8). To reduce postoperative pain, previous studies have proposed optimal ligation heights above the dentate line, ranging from 2 to 5 mm (9) and 7 mm (10) to 1 cm (11) and even approximately 2 cm (12). However, ligation too far above the dentate line may compromise efficacy by inadequately targeting the hemorrhoidal cushion. Moreover, interindividual variability in anal canal length limits the reliability of distance-based references. Therefore, a new and anatomically reliable operative landmark is needed.

In our previous clinical observations and related studies (13), a distinct mucosal demarcation line (MDL) was frequently visible under endoscopic white-light imaging. This line is located above the dentate line and is characterized by a sharp contrast in mucosal color on either side. Yamataka et al. similarly identified this MDL when the anal canal mucosa was pulled outward during transanal pull-through procedures for Hirschsprung’s disease (14). Several studies have suggested that MDL may correspond to the upper border of anal transitional zone (ATZ) (15). ATZ is a transitional region between the anal canal and the rectum, with its upper border located approximately 3–20 mm above the dentate line (16). Intraepithelial free nerve endings and sensory corpuscles within ATZ detect pain, temperature, and touch (17), and play an essential role in maintaining the defecation reflex (18). Complete excision of ATZ may result in sensory impairment and even sensory anal incontinence (19). Several studies have revealed that preservation of ATZ during anal canal surgery is associated with better long-term anal function and fewer complications (19, 20).

However, the proposed correspondence between MDL and the upper border of ATZ has not been histologically validated, and its clinical utility as an operative landmark for endoscopic internal hemorrhoid therapy remains unexplored. Accordingly, this research aimed to histologically clarify their correlation and to preliminarily assess the efficacy, safety, and potential mechanisms of MDL-guided endoscopic treatment for internal hemorrhoids.

## Materials and methods

### Ethical approval

This single-center study was performed at the Department of Gastroenterology, Xi’an NO.3 Hospital, from September 2025 to April 2026. The study protocol was approved by the Xi’an No.3 Hospital Ethics Committee and registered at chictr.org.cn (ChiCTR2500107924). Written informed consent was obtained from all individuals before enrollment.

### Endoscopic identification of MDL

Adults aged 18–80 years who were scheduled for gastrointestinal endoscopy for health examination or screening purposes were enrolled in this part of the study. The exclusion criteria were as follows: Anorectal disease (anal malignancy, anal fistula, proctitis, or inflammatory bowel disease), a history of hemorrhoidectomy or RBL, and severe rectal prolapse.

With participants in the left lateral decubitus position, an experienced endoscopist performed cap-assisted endoscopy (Olympus). The endoscope was retroflexed in the rectum, and gentle air insufflation was used to fully expose the anorectum. Anorectal landmarks, including the anal columns, anal crypts, anal valves, dentate line, and MDL, were identified under both white-light imaging and narrow band imaging (NBI). Using the outer diameter of the endoscope (10.0 mm) and the fully opened biopsy forceps (5 mm) as reference scales, the distance between MDL and the dentate line was measured at three equidistant points approximately 120°apart around the circumference. The mean, maximum, and minimum distances were recorded.

### Histological confirmation of MDL

**Figure 1:**
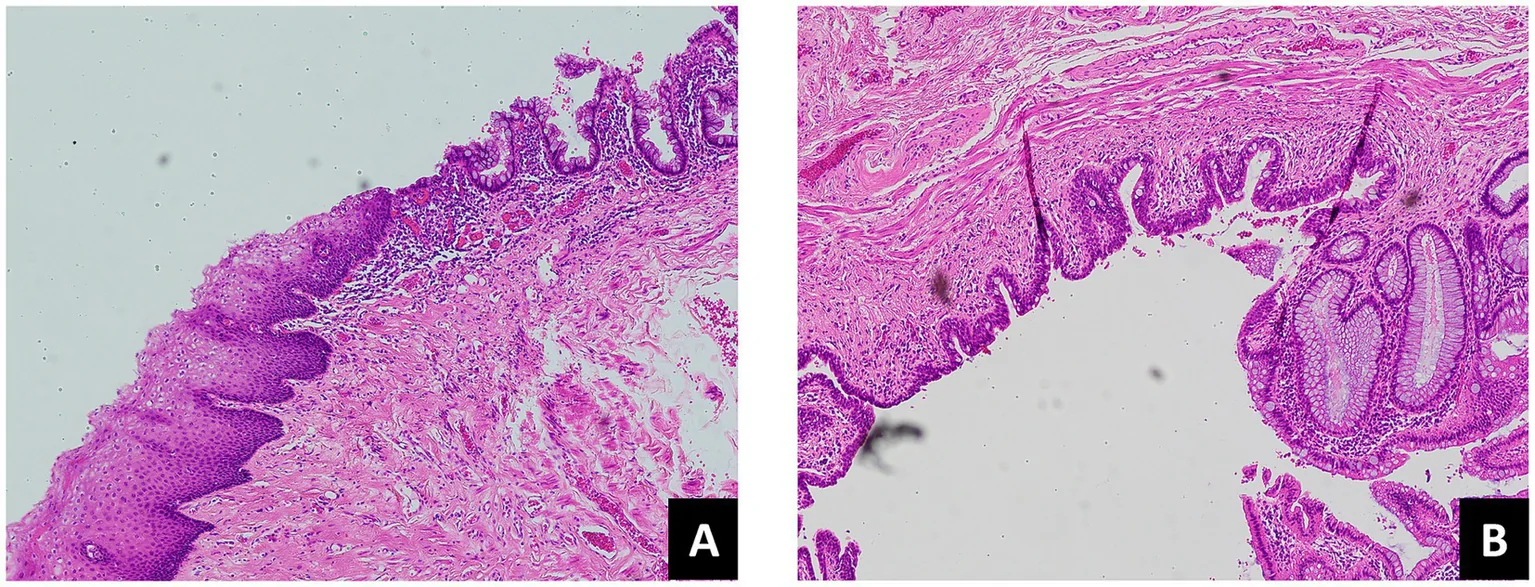
Epithelial transitions in anal canal specimens: junction of simple columnar epithelium with stratified squamous epithelium **(A)**; junction of simple columnar epithelium with stratified columnar epithelium **(B)**.

In three participants, mucosal biopsy specimens (1–2 mm) were acquired from three locations: One straddling MDL and two located 0.5 cm above and below MDL. Specimens were paraffin-embedded, serially sectioned, and stained with hematoxylin and eosin (H&E) to evaluate epithelial characteristics. Based on our previous study of complete longitudinal anal canal sections, simple columnar epithelium most commonly adjoined continuous stratified squamous epithelium (Figure 1A) and less frequently abutted stratified columnar epithelium (Figure 1B), align with the findings of Tanaka et al. (21). Accordingly, histological validation of the upper border of ATZ was defined as the transition from simple columnar to stratified squamous epithelium, with occasional stratified columnar epithelium extending from anal crypts.

### Collection and processing of anal canal specimens

Five intact and macroscopically normal anal canal specimens were obtained from patients undergoing abdominoperineal resection for rectal cancer. After transection at the dentate line, 13 longitudinal tissue blocks (0.5 cm thick) were collected from 2.0 cm above the dentate line and an additional 13 blocks from 2.0 cm below it. Tissues were fixed in 10% neutral buffered formalin and embedded in paraffin. Serial sections (3 μm) were cut longitudinally, placed on gelatin-coated slides, dried, and processed for H&E staining or immunohistochemistry.

H&E-stained sections were used to identify the mucosal, submucosal, and muscular layers of the anal canal. In sections above the dentate line, MDL and hemorrhoidal cushions, composed of vascular plexuses and loose connective tissue, were identified, and their spatial relationship was assessed.

### Immunohistochemistry

Immunohistochemical staining was performed using PGP9.5 and S100 antibodies to label nerve fibers and Schwann cells, respectively. Sections were deparaffinized, rehydrated through graded alcohol, and subjected to heat-induced antigen retrieval in 10× Tris-EDTA buffer. Following blocking endogenous peroxide activity with 3% hydrogen peroxide and nonspecific binding with 5% bovine serum albumin, sections were incubated overnight at 4 °C with primary antibodies (anti-PGP9.5, 1:100, Affinity Biosciences; anti-S100, ready-to-use, MXB Biotechnologies). Sections were then incubated with secondary antibody (mouse/rabbit, Dako, and SM802) at room temperature, followed by chromogenic development with 3,3′-diaminobenzidine. The sections were subsequently counterstained with hematoxylin, differentiated in 1% acid alcohol, blued under running tap water, dehydrated through graded ethanol, cleared in xylene, and mounted with neutral resin.

The area ratio of PGP9.5-positive free nerve endings was quantified in the ATZ epithelium above the dentate line as well as in the non-keratinized and keratinized squamous epithelium below it. Images were captured at ×400 magnification, and the ratio of PGP9.5-positive area to total epithelial area was calculated employing ImageJ software, averaged across five randomly maintained high-power fields per section. In S100-stained sections, sensory corpuscles were verified and examined within the mucosal and submucosal layers.

### Clinical study

Ninety-three participants with symptomatic internal hemorrhoids were enrolled to receive MDL-guided endoscopic treatment. The inclusion criteria were as follows: adults aged 18–80 years with symptomatic Grade I-III internal hemorrhoids and selected Grade IV internal hemorrhoids (declining surgery or with surgical contraindications) suitable for endoscopic treatment. The exclusion criteria were as follows: Mixed or external hemorrhoids; internal hemorrhoids complicated by incarceration, thrombosis, ulceration, or infection; severe cardiac, pulmonary, hepatic, or renal failure; active perianal infection, anal fistula, active inflammatory bowel disease or other organic colorectal disease; severe coagulopathy; known hypersensitivity to sclerosants; psychiatric diseases precluding informed consent; incomplete data or loss to follow-up. The treatment modality (sclerotherapy or RBL) was obtained based on hemorrhoid grade, symptoms, technical feasibility, contraindications, and patient preference.

**Figure 2:**
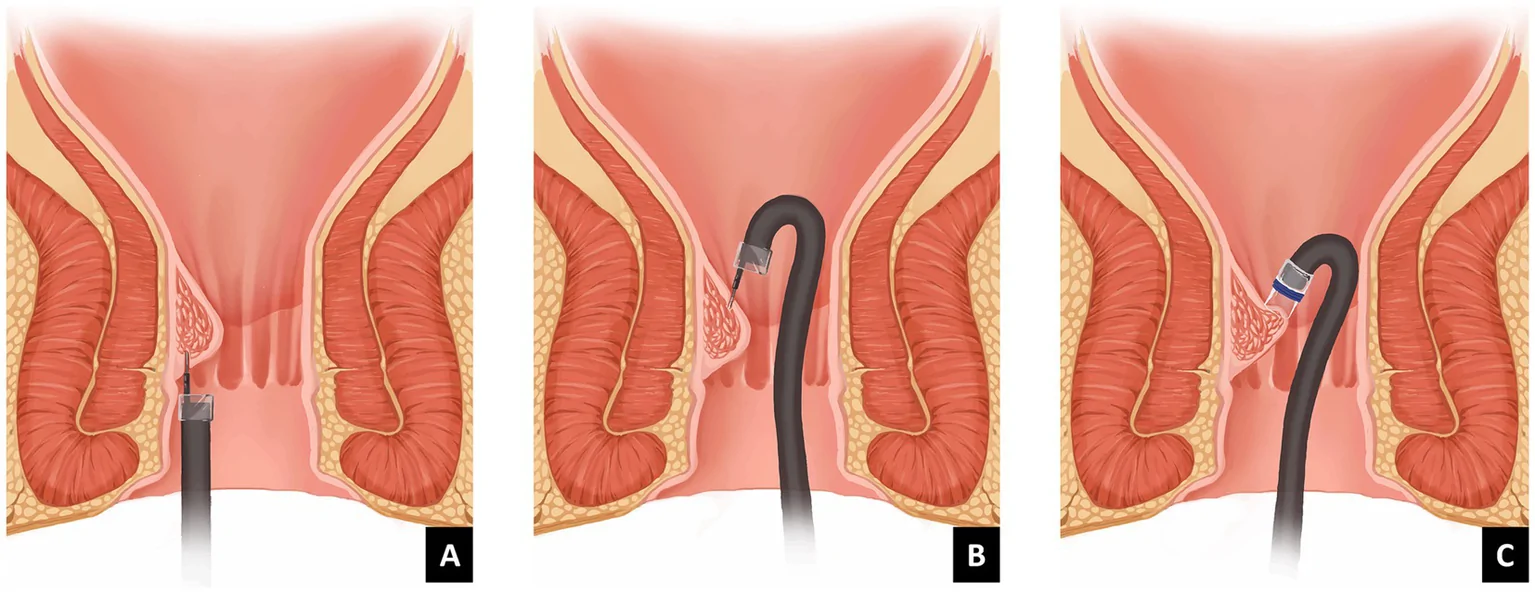
MDL-guided endoscopic treatment of internal hemorrhoids: sclerotherapy in the forward view **(A)** and retroflexed view **(B)**; rubber band ligation **(C)**.

Sclerotherapy was performed employing an upper endoscope (Pentax, Japan) fitted with a straight transparent distal cap. The procedure was performed in either the forward or retroflexed view, depending on the anatomical conditions of the anal canal. In the forward view, the injection site was selected midway between the dentate line and MDL, and the needle was advanced obliquely in the oral direction for submucosal injection (Figure 2A). In the retroflexed view, a site 2–3 mm oral to MDL was selected, and the needle was advanced obliquely in the anal direction (Figure 2B). Undiluted polidocanol mixed with methylene blue was injected at 0.6–1.0 mL per point across 3–5 points, with a total dose not reaching 10 mL per session. The needle was withdrawn slowly during injection to create a continuous submucosal sclerosing column.

RBL was performed employing an upper endoscope (Pentax, Japan) equipped with a band ligator device. The rubber band was deployed immediately above the level of MDL, ligating the mucosa and submucosa on the oral side (Figure 2C). The band number and ligation sites were individualized according to hemorrhoid characteristics, with adequate spacing between adjacent ligation sites and avoidance of continuous circumferential ligation. A maximum of five ligation sites were treated per session. Adequate elevation of the hemorrhoidal cushion was confirmed after each ligation.

An internal-hemorrhoid symptom questionnaire was completed before treatment and at 2, 4, 8, and 12 weeks, as well as 6 months after treatment. Part 1 assessed hematochezia and prolapse. Treatment response was classified as cured (complete resolution of symptoms without recurrence on endoscopy), improvement (partial symptom relief with downgraded hemorrhoid grade), or no response (persistent or worsened symptoms without significant endoscopy change). Effective rate = (cure + improved)/total cases × 100%; cure rate = cure/total cases × 100%. Recurrence was defined as the reappearance of hematochezia or prolapse that persisted for more than 1 week. Part 2 assessed perianal discomfort (perianal discomfort, moisture, anal fullness, pain, and a sense of incomplete evacuation), as well as defecatory disorders (difficult or obstructed defecation, constipation, narrow or unformed stools, and increased stool frequency). Each symptom was scored as 2 (ineffective), 1 (improved), or 0 (cured), and domain scores were calculated as the sum of individual item scores.

All complications were reported and classified as minor (mild postoperative pain, anal fullness, minor bleeding, or self-limited urinary retention) or major (severe pain requiring intervention, infection, fecal incontinence, anal stricture, or thrombosis).

### Statistical analysis

Statistical analyzes were performed using the Statistical Package for the Social Sciences software (version 26.0). Categorical variables are presented as frequencies and percentages, and continuous variables as mean ± standard deviation for normally distributed data or median (interquartile range) for non-normally distributed data. Normally distributed data were compared using the *t*-test, while non-normally distributed data were compared using nonparametric tests. Categorical data were compared using *χ*^2^ or Fisher’s exact test. A *p* < 0.05 indicated statistical significance.

## Results

### Endoscopic identification of MDL and its relationship to the dentate line

A total of 207 participants underwent endoscopic examination and were included in the final analysis: 112 men and 95 women, aged 22–77 years (median age: 53 years).

**Figure 3:**
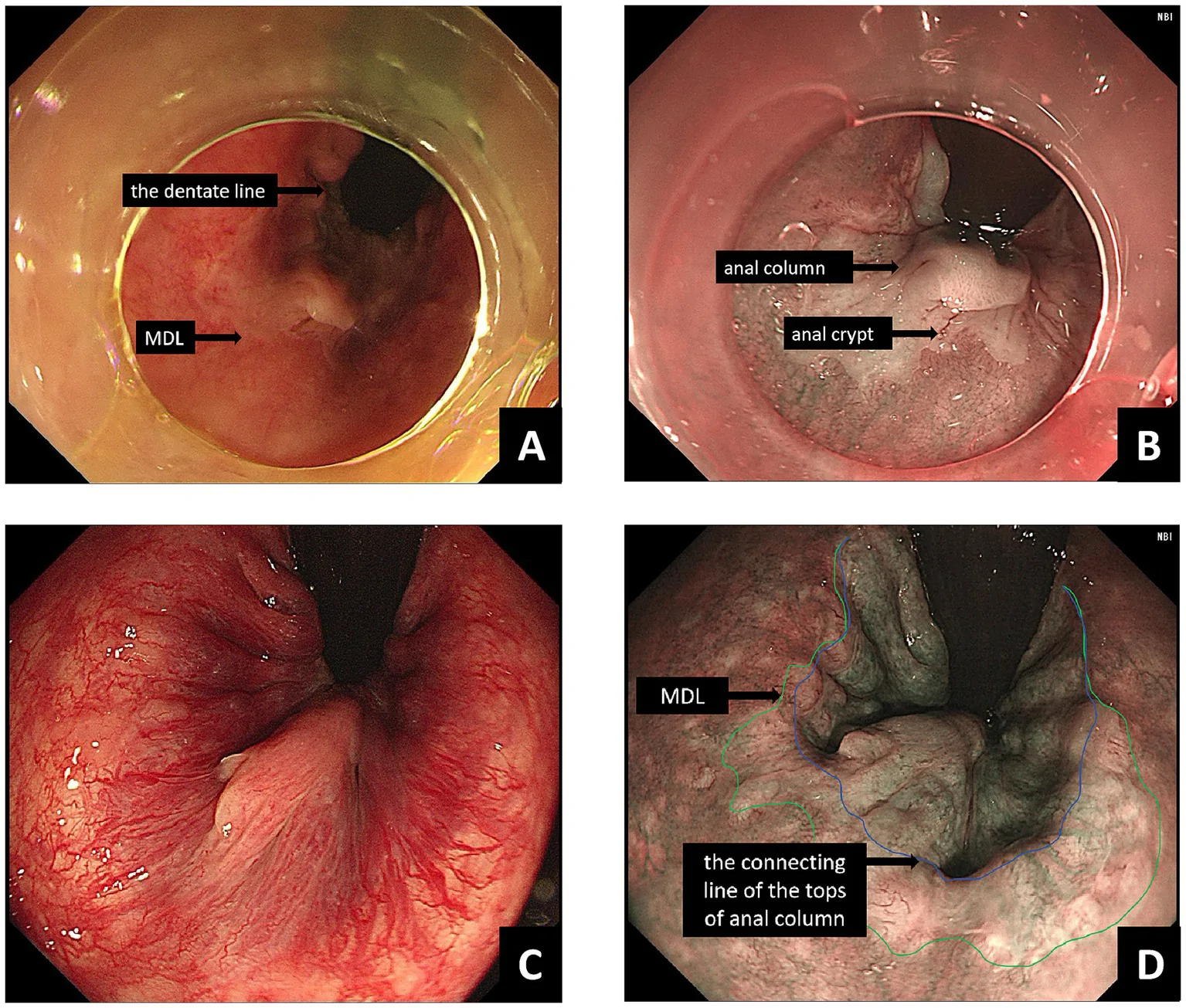
MDL in the adult anal canal: MDL is clearly visible under white-light imaging **(A)** and NBI **(B)**. MDL is indistinct under white-light imaging **(C)** but clearly visualized under NBI **(D)**.

Under white-light imaging, typical anatomical structures of the anal canal and rectum, including anal crypts, columns, valves, and the dentate line, were clearly identified (Figures 3A,B). MDL was observed above the dentate line, with morphology ranging from smooth and curved to irregular and coastline-like patterns (Figure 3A). The mucosa on the anal side of MDL appeared pale pink, while the mucosa on the oral side appeared red. Under NBI, the mucosa on the anal side of MDL seemed to be light green, with bluish-green vessels visible beneath the surface, densely arranged in radial orientations and partially extending across MDL (Figures 3B,D). In contrast, the mucosa on the oral side appeared brownish, with sparsely distributed bluish-green, arborizing vessels.

MDL was identified in 69.08% of participants (143/207) under white-light imaging (Figures 3A,C), and this rate elevated significantly to 96.14% (199/207) under NBI (*p* < 0.001, *χ*^2^ test) (Figures 3B,D). The visualization of the top of the anal columns was influenced by insufflation, leading to positional variation. Their connecting line did not coincide with MDL (Figure 3D). The mean distance from MDL to the dentate line was 8.18 ± 2.96 mm. Considerable intraindividual variability was observed, with an average range of 3.16 ± 1.93 mm. This distance indicated a non-significant association with sex, age, height, or BMI (*p* > 0.05).

### Epithelial characteristics of MDL

**Figure 4:**
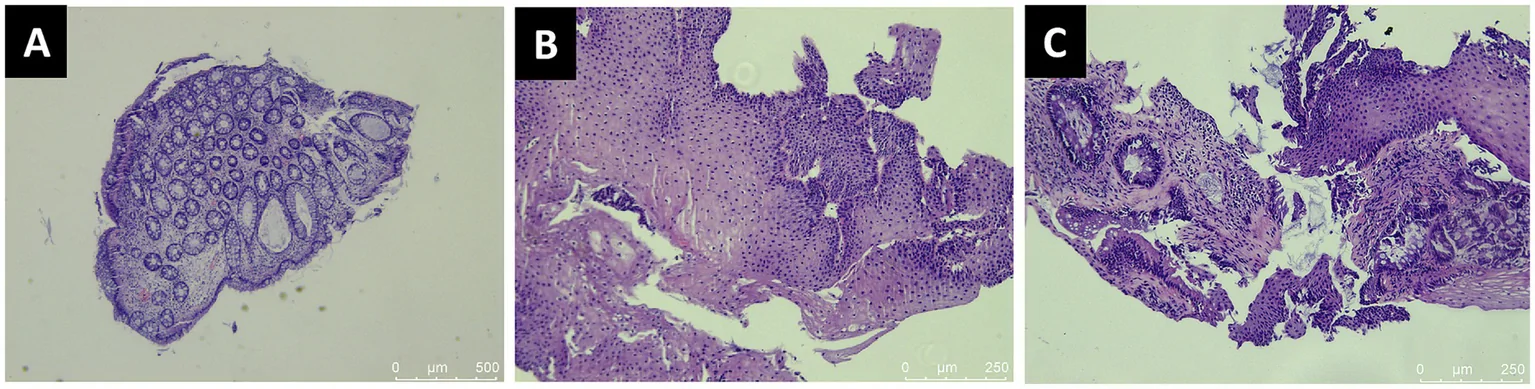
Epithelial characteristics of MDL. Above MDL: simple columnar epithelium **(A)**. Below MDL: stratified squamous epithelium **(B)**. At MDL: columnar epithelium sharply transitions to stratified squamous epithelium **(C)**.

Biopsy specimens obtained 0.5 cm above MDL indicated simple columnar epithelium (Figure 4A). At MDL, the epithelium transitioned abruptly from simple columnar epithelium on the oral side to stratified squamous epithelium on the anal side (Figure 4C). Specimens obtained 0.5 cm below MDL comprised stratified squamous epithelium (Figure 4B). These findings confirm that MDL represents the upper border of ATZ.

### Location of the anal cushions and neural distribution

**Figure 5:**
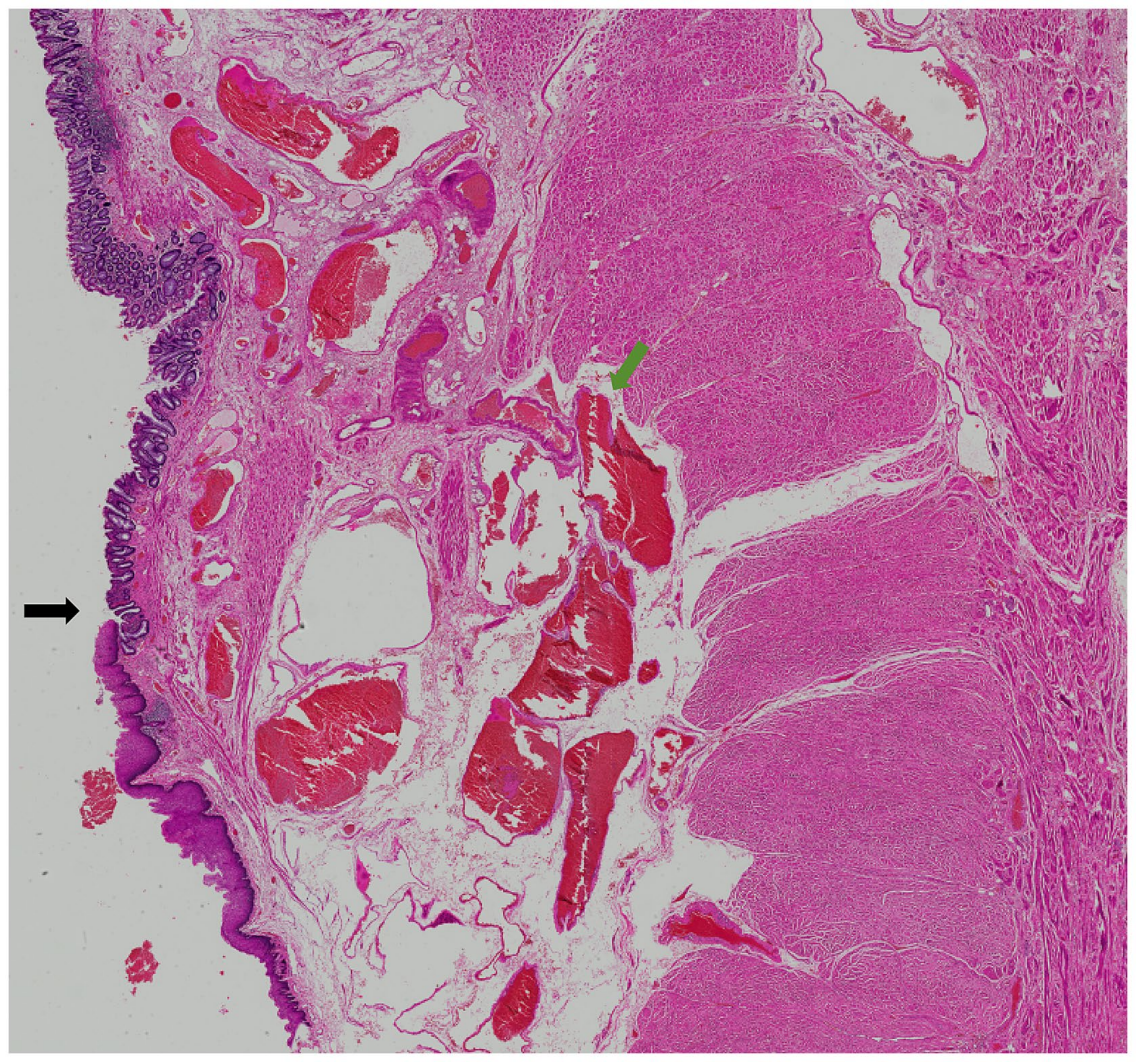
Location of anal cushions (green arrow) and MDL (black arrow).

In all 13 H&E-stained sections of specimens obtained above the dentate line, MDL was identifiable and represented the upper border of ATZ. Anal cushions were observed in nine sections and were composed of arteriovenous plexus and connective tissue overlying the internal anal sphincter. In all cases, MDL was positioned below both the anal cushion tissue and the upper margin of the internal anal sphincter (Figure 5). The mean length of the anal cushion was 16.21 ± 6.01 mm, extending an average of 6.81 ± 4.21 mm beyond MDL. Below the dentate line, the mucosa consisted of continuous non-keratinized and keratinized stratified squamous epithelium, and the submucosa contained connective tissue, small vessels, adipose tissue, and occasional hair follicles.

**Figure 6:**
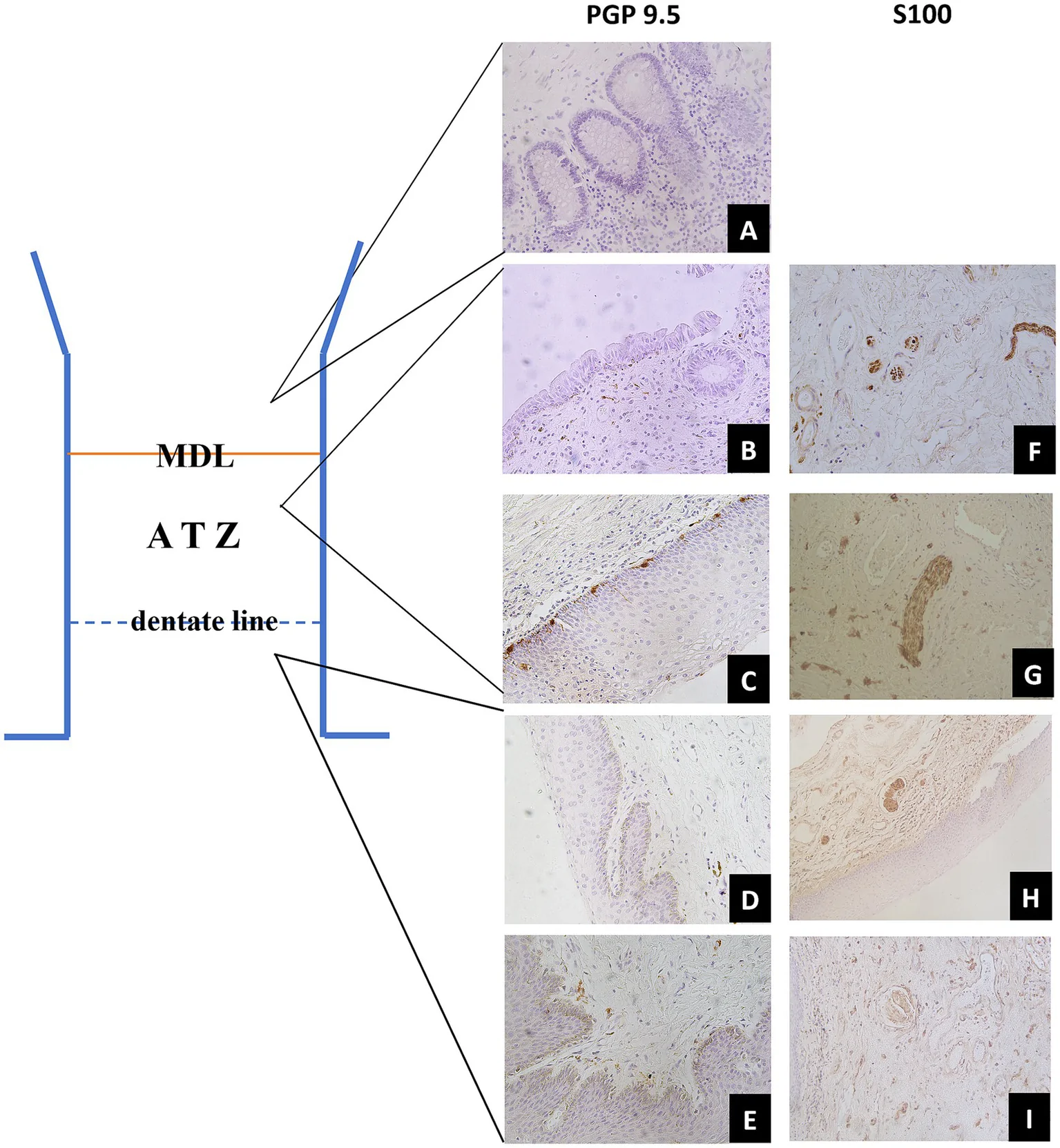
Mucosa and neural distribution of the anal canal: no free nerve endings in simple columnar epithelium **(A)**. Free nerve endings in stratified columnar epithelium **(B)**. Free nerve endings and Merkel cells in stratified squamous epithelium **(C)**. Free nerve endings in non-keratinized **(D)** and keratinized **(E)** stratified squamous epithelium. Submucosal nerve fibers and bundles in all regions **(F,G)**. Krause corpuscles **(I)** and Meissner corpuscles **(H)** in the submucosa below the dentate line.

Nerve tissue was observed in the anal canal both above and below the dentate line. In specimens obtained above the dentate line, no PGP9.5-positive free nerve endings were detected within the simple columnar epithelium (Figure 6A), whereas nerve fibers were present in the mucosa and submucosa. Within ATZ below MDL, PGP9.5-positive free nerve endings were found in both stratified squamous and stratified columnar epithelium, primarily at the basal layer, with scattered Merkel cells (Figures 6B,C). Nerve fibers and bundles traversed the connective tissue and vascular plexus in the submucosa (Figures 6F,G), but no sensory corpuscles were identified. In specimens obtained below the dentate line, PGP9.5-positive free nerve endings were also detected in both non-keratinized and keratinized stratified squamous epithelium (Figures 6D,E), at significantly higher area ratios than those observed in the epithelium above the dentate line (Table 1). Additionally, Meissner corpuscles (Figure 6H), Krause corpuscles (Figure 6I), as well as scattered nerve fibers and bundles were observed in the submucosa (Figures 6F,G).

**Table 1:** PGP9.5-positive intraepithelial free nerve endings area ratio in different regions.

Anatomical location	Epithelial type	PGP9.5-positive intraepithelial free nerve endings area ratio (%)	*P*
Above the dentate line	ATZ	0.58±0.37	
Below the dentate line	Non-keratinized	1.10±0.75	0.038*
	Keratinized	1.64±0.58	<0.001*

^*^Compared with the area ratio above the dentate line.

### Clinical efficacy and safety of MDL-guided endoscopic therapy

Ninety-three patients with symptomatic internal hemorrhoids were enrolled, including 61 receiving sclerotherapy and 32 undergoing RBL. Demographic and clinical characteristics of the two treatments are indicated in Table 2. All patients in the present study underwent a single treatment session. In the sclerotherapy group, the mean sclerosant volume was 5.72 ± 1.97 mL. The mean number of rubber bands applied was 4.00 ± 0.72 in the RBL group.

**Table 2:** Demographic and clinical characteristics.

Characteristic	Sclerotherapy	RBL	*P*
Sex(male/female)	36/25	23/9	0.221
Age(years)	52.00±13.58	52.06±10.04	0.980
Hemorrhoid grading (I/II/III/IV)	37/23/1/0	0/16/15/1	<0.001

Patients receiving sclerotherapy had predominantly Grade I-II internal hemorrhoids, including 55 with hematochezia and 24 with prolapse. Overall effective rates at 2 weeks and 6 months were 95.08 and 91.80%, respectively, with cure rates of 91.80 and 88.52% (Table 3). Hematochezia completely resolved in 92.73% (51/55), 96.36% (53/55), 94.55% (52/55), 92.73% (51/55), and 89.09% (49/55) of patients at 2, 4, 8, and 12 weeks, and 6 months, respectively, while prolapse completely resolved in 95.83% (23/24), 95.83% (23/24), 95.83% (23/24), 100.00% (24/24), and 95.83% (23/24). The cumulative six-month recurrence rates were 5.45% for hematochezia and 4.17% for prolapse, with an overall recurrence of 6.56%.

**Table 3:** Efficacy and recurrence of sclerotherapy and RBL for internal hemorrhoids.

Treatment	Outcome	2 weeks	4 weeks	8 weeks	12 weeks	6 months
Sclerotherapy (*n* = 61)	Effective	58 (95.08%)	60 (98.36%)	58 (95.08%)	58 (95.08%)	56 (91.80%)
	Cure	56 (91.80%)	58 (95.08%)	57 (93.44%)	57 (93.44%)	54 (88.52%)
	Recurrence for hematochezia (55)	/	1	0	2	0
	Recurrence for prolapse (24)	/	0	0	0	1
RBL (*n* = 32)	Effective	30 (93.75%)	30 (93.75%)	29 (90.63%)	30 (93.75%)	30 (93.75%)
	Cure	28 (87.50%)	25 (78.13%)	26 (81.25%)	29 (90.63%)	29 (90.63%)
	Recurrence for hematochezia (24)	/	1	0	0	0
	Recurrence for prolapse (32)	/	0	1	0	0

In patients undergoing RBL, 24 indicated with hematochezia, and all 32 presented with prolapse, with the majority having Grade II-III internal hemorrhoids. At 2 weeks and 6 months, overall effective rates were both 93.75%, with cure rates of 87.50 and 90.63%, respectively. The cure rates for hematochezia at 2, 4, 8, and 12 weeks and 6 months were 87.50% (21/24), 83.33% (20/24), 87.50% (21/24), 100.00% (24/24), and 100.00% (24/24). The cure rates for prolapse at the same time points were 96.88% (31/32), 90.63% (29/32), 90.63% (29/32), 93.75% (30/32), and 90.63% (29/32). By 6 months, the cumulative recurrence rates were 4.17% for hematochezia and 3.13% for prolapse, and the overall recurrence rate was 6.25% (Table 3).

Preoperatively, 87.10% of patients with internal hemorrhoids (81/93) experienced perianal discomfort. Among the 56 patients with prolapse, 53 (94.64%) reported perianal discomfort, compared with 28 of 37 patients (75.68%) without prolapse, indicating a statistically significant difference (*p* = 0.011, Fisher’s exact test). Defecatory disorders were present in 66 patients (70.97%) before treatment. Although the proportion of patients with prolapse having defecatory disorders was higher among those with prolapse than among those without prolapse (75% vs. 64.86%), this difference was not statistically significant (*p* = 0.292). Among patients receiving sclerotherapy, 49 reported perianal discomfort, and 41 reported defecatory disorders. In the RBL group, all 32 patients reported perianal discomfort, and 16 reported defecatory disorders. At 2 weeks following treatment, sclerotherapy decreased the perianal discomfort scores from 4 (2, 6) to 0 (0, 0) (*p* < 0.001) and the defecatory disorders scores from 2 (0, 4) to 0 (0, 0) (*p* < 0.001). Similarly, in the RBL group, the median perianal discomfort score decreased from 4 (2, 6) to 0 (0, 2) (*p* < 0.001), and the defecatory disorders score decreased from 4 (2, 4) to 0 (0, 0) (*p* < 0.001).

**Figure 7:**
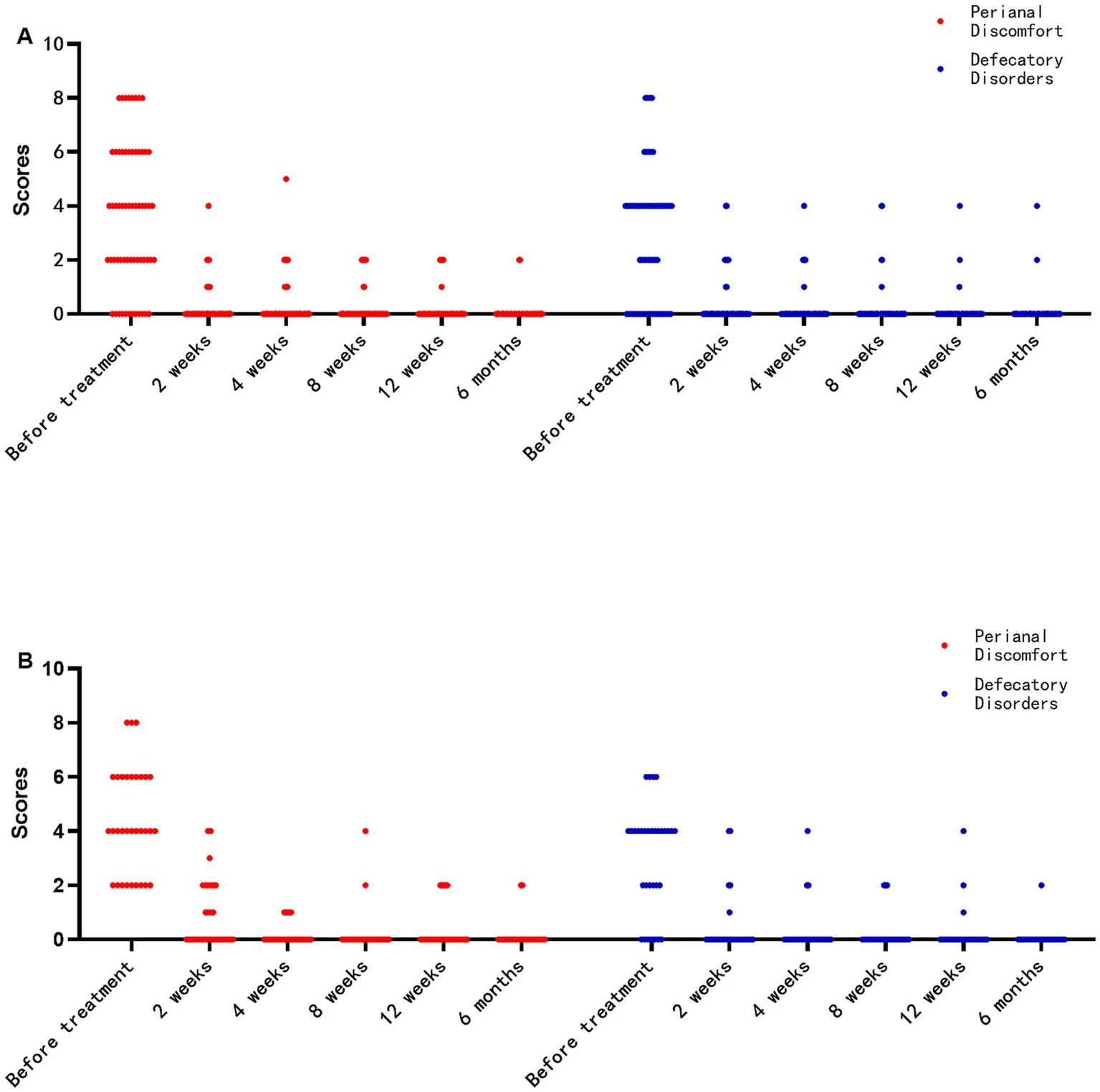
Perianal discomfort and defecatory disorders scores following sclerotherapy **(A)** and RBL **(B)**.

Across follow-up visits, non-significant differences were observed in effective or cure rates (Table 3) and perianal discomfort or defecation disorder scores (Figure 7) after sclerotherapy or RBL (all *p* > 0.05).

Major complications, including severe pain, infection, fecal incontinence, or thrombosis, were not observed in the treatment group, and no cases of anal stricture occurred during follow-up. However, minor complications occurred in some patients. Within 24 h following sclerotherapy, 6.56% (4/61) of patients reported new perianal discomfort, including mild pain and anal fullness, compared with 18.75% (6/32) of those undergoing RBL. All symptoms were resolved within 1 week with symptomatic management. Minor postoperative bleeding was reported in 3.28% (2/61) of sclerotherapy patients, likely associated with hard stools. In 9.38% (3/32) of RBL patients, it also occurred primarily 5–12 days following treatment, probably due to band detachment. Additionally, two patients (6.25%) after RBL experienced urinary retention on the first postoperative day, which was resolved by the next day following warm compress therapy.

## Discussion

The upper border of ATZ is readily identifiable *in vivo* and can be directly visualized endoscopically. Prior studies of ATZ relied significantly on gross or resected specimens that may undergo autolysis during processing (22), leading researchers to consider identification of the upper border of ATZ challenging without Alcian blue staining. A distinct MDL was consistently observed above the dentate line in both our previous work and this current study. Our biopsy findings confirmed sharply varying epithelial types on either side of MDL, verifying for the first time that MDL represents the upper border of ATZ. In our research, the upper border of ATZ was more readily identified under NBI compared with under white-light imaging. It is likely because NBI improves the contrast between squamous and columnar epithelium by retaining specific wavelengths (23). Previous studies of Barrett’s esophagus and gastric squamous metaplasia (23, 24) also reported similar squamo-columnar junction visualization.

We found that the mean distance from MDL (the upper border of ATZ) to the dentate line was 8.18 mm, which aligns with the previously reported value of approximately 8.4 mm (16). Several studies have proposed that the upper border of ATZ corresponds to the anorectal line (14, 15). However, others have disputed this association (13), due to ongoing controversy over the definition of the anorectal line. Currently, the anorectal line is commonly regarded as the upper boundary of the surgical anal canal, corresponding to the upper margin of the internal anal sphincter or to the level at which the rectum begins to expand into the pelvic bowl (25). However, our study indicated that the upper border of ATZ is consistently located below the upper margin of the internal anal sphincter. Other studies have defined the upper boundary of the surgical anal canal as the line connecting the tops of the anal columns (26); however, this line did not coincide with the upper border of ATZ in our observation. These results indicated that the upper border of ATZ does not correspond to the surgically defined anorectal line. Additionally, some studies have considered the anorectal line synonymous with the Herrmann line (20). This term originates from Herrmann, who, in 1880, described the boundary where the anal canal mucosa transitions to rectal mucosa as the anorectal line during his observations (27). Based on this original definition, the upper border of ATZ essentially corresponds to the Herrmann line. However, some authors have used the term anorectal line in its surgical sense while applying it to mucosal transitions, without distinguishing between the anatomical and epithelial definitions, resulting in conceptual misuse and confusion (28). Given the widespread clinical acceptance of the surgical anal canal concept, the term anorectal line should refer exclusively to its upper boundary. To avoid confusion, the upper border of ATZ should not be referred to as the anorectal line. Furthermore, the term ‘Herrmann line’ is not recommended, as it is now uncommon in recent literature and clinical practice.

In this study, we observed that both sclerotherapy and RBL with the upper border of ATZ as a reference achieved a favorable therapeutic effect in patients with internal hemorrhoids, possibly associated with the anatomical position of the hemorrhoidal cushions. A prior research of sclerotherapy for Grade I internal hemorrhoids recorded a success rate of 88% at 12 weeks (29). Salgueiro et al. reported an overall efficacy of 93.4% for sclerotherapy in Grade I–III internal hemorrhoids (30). Another survey indicated success rates of 68.3% at 1 week and 95.6% at 1 year for sclerotherapy in Grade I internal hemorrhoids, with a bleeding recurrence rate of 12% (31). Gallo et al. reported a recurrence rate of approximately 16.1% following sclerotherapy (32). In our study primarily evaluating sclerotherapy for Grade I–II internal hemorrhoids, the effective rate remained above 90% from 2 weeks to 6 months after treatment, with a bleeding recurrence rate of 5.45%. Previous studies of RBL for Grade II–IV internal hemorrhoids reported effectiveness rates ranging from approximately 88 to 95.4% (9, 33). However, recurrence rates suggested considerable variability: Some studies documented rates as high as 49% (34), and others reported 2-year recurrence rates of 11–12% (33, 35). Our study of Grade II–III internal hemorrhoids demonstrated a 2-week effective rate of 93.75% and an overall recurrence rate of 6.25%. These results were highly consistent with previous reports, and the low recurrence rate suggested a sustained therapeutic effect during the 6-month follow-up. Our histological analysis revealed that ATZ covered lower portion of the anal cushions. Since internal hemorrhoids are displaced and changed anal cushions, the injection orientations were adjusted according to the endoscopic view (forward or retroflexed) to facilitate delivering the sclerosant into the target submucosal hemorrhoidal tissues, and to minimize the unnecessary injury to non-target tissues. In RBL, ligation was conducted on the oral side of ATZ upper border. Despite being above the dentate line and not fully including the hemorrhoidal cushion, ligation at this level still captures its upper portion, effectively interrupting local blood flow and inducing fibrosis, thereby decreasing hemorrhoidal size and stabilizing prolapsed mucosa.

We observed that perianal discomfort and defecatory disorders were improved, possibly associated with the repositioning of ATZ mucosa, which contains abundant intraepithelial free nerve endings. Additionally, multiple previous studies reported alleviation of perianal itching and pain following endoscopic treatment (10, 29). We speculated that these improvements might be related to the neural distribution within ATZ mucosa. In the present study, PGP9.5 and S100 immunostainings were introduced to evaluate peripheral neural and neural-associated structures. Although no sensory corpuscle was observed within the submucosa, intraepithelial free nerve endings were identified above the dentate line, consistent with previous reports (17, 36), suggesting that sensory neural components were present within the ATZ mucosa. Despite their lower area ratio than that in the mucosa below the dentate line, these intraepithelial free nerve endings can directly sense luminal stimuli and mediate sensations of temperature, pain, itch, and light touch. Therefore, enlargement and prolapse of the hemorrhoidal cushions displace the ATZ mucosa from its normal position in patients with internal hemorrhoids, likely contributing to perianal discomfort and abnormal defecatory sensations. This may explain why patients with prolapse in our research reported more perianal discomfort. Following sclerotherapy or RBL, the preserved ATZ mucosa is repositioned, alleviating perianal discomfort and normalizing defecatory sensations.

In our study, endoscopic treatment guided by the upper border of ATZ was safe, with no major complications observed. In previous reports, complications associated with sclerotherapy have generally been mild, with approximately 8.0% of patients experiencing transient self-limiting pain (37) and a postoperative bleeding rate of 4.8% (30). Our results indicated that the incidence of discomfort and bleeding following sclerotherapy was 6.56 and 3.28%, respectively, suggesting a favorable safety profile. Conversely, RBL has often been associated with higher complication rates due to necrosis and subsequent detachment of the hemorrhoid. A study assessing complications following RBL reported that 90% of patients experienced postoperative pain, with 7% continuing to report moderate to severe pain at 1 week, and a bleeding rate of 24% at 1 week (38). Other studies have documented postoperative pain rates of 60% and bleeding rates of 15% (39). In the present study, postoperative discomfort and bleeding following RBL occurred in 18.75 and 9.38% of patients, respectively, indicating an acceptable safety. Traditionally, RBL has been performed with reference to the dentate line because the region below it is considered to be richly innervated by somatic nerves. However, Chan et al. reported that ATZ is also innervated by the somatic pudendal nerve (40), rather than by visceral nerves. Using the upper border of ATZ as the reference and placing ligation above it may therefore reduce stimulation of somatic nerves within ATZ and decrease postoperative complications, especially pain.

This study has several limitations. The efficacy evaluation lacked a control group due to ethical considerations and relied on before-and-after comparisons, which preclude direct conclusions regarding superiority or inferiority relative to other therapeutic strategies. Moreover, multicenter studies with larger sample sizes and longer follow-up periods are needed to further validate these results.

In conclusion, the upper border of ATZ is readily identifiable endoscopically and can serve as an anatomical landmark for endoscopic treatment of internal hemorrhoids, with favorable clinical outcomes and an acceptable safety profile. These effects might be associated with its anatomical correlation with the hemorrhoidal cushions and the preservation of ATZ.

## Data Availability

The raw data supporting the conclusions of this article will be made available by the authors, without undue reservation.
